# Epithelial Cell Dysfunction in Pulmonary Fibrosis: Mechanisms, Interactions, and Emerging Therapeutic Targets

**DOI:** 10.3390/ph18060812

**Published:** 2025-05-28

**Authors:** Jing Wang, Jie Chao

**Affiliations:** 1 Department of Physiology, School of Medicine, Southeast University, Nanjing 210009, China; wangjing2016@seu.edu.cn; 2 Jiangsu Provincial Key Laboratory of Critical Care Medicine, Zhongda Hospital, School of Medicine, Southeast University, Nanjing 210009, China

**Keywords:** pulmonary fibrosis (PF), epithelial dysfunction, epithelial–mesenchymal transition (EMT), cell heterogeneity, antifibrosis strategies

## Abstract

Pulmonary fibrosis (PF) is a progressive and fatal interstitial lung disease characterized by chronic epithelial injury and excessive deposition of extracellular matrix (ECM) driven by dysregulated repair. Increasing evidence has shown that epithelial cell dysfunction plays a key role in PF, involving epithelial–mesenchymal transition (EMT), chronic oxidative stress, disruption of epithelial–immune interactions, and promoting pathological remodeling. Single-cell analyses have identified functionally distinct subpopulations of type 2 alveolar (AT2) cells with pro-fibrotic potential. Epithelial cells exhibit metabolic and epigenetic alterations during PF, which provide new approaches for therapeutic targets. This review summarizes the molecular mechanisms driving epithelial dysfunction in fibrosis progression, with a focus on key regulatory pathways, including transforming growth factor-beta (TGF-β), Wnt, and Notch signaling pathways, as well as miRNA-mediated networks. We also explored emerging epithelial-targeted therapies, ranging from FDA-approved agents (pirfenidone, nintedanib) to experimental inhibitors targeting Galectin-3 and Wnt/β-catenin, providing insights into precision anti-fibrosis strategies for clinical translation.

## 1. Introduction

Tissue fibrosis is a pathological process characterized by the excessive deposition of extracellular matrix (ECM) components, resulting in structural disruption and functional impairment of affected tissues [1]. This process is commonly observed in various chronic diseases, including liver fibrosis, pulmonary fibrosis (PF), and renal fibrosis. The progression of fibrosis is closely linked to inflammation, ECM remodeling, and fibroblast activation, leading to irreversible tissue scarring and organ dysfunction [2]. In particular, PF, notably idiopathic pulmonary fibrosis (IPF), is marked by progressive dyspnea, non-productive cough, and an irreversible decline in lung function, which often results in respiratory failure [3]. The median survival following diagnosis is typically 3 to 5 years [4]; however, current therapies, such as pirfenidone and nintedanib, significantly slow the annual decline in forced vital capacity (FVC) and reduce the risk of acute exacerbations in IPF patients [5,6]. Still, neither agent can halt or reverse established fibrotic remodeling. Real-world and systematic evaluations have also demonstrated sustained tolerability and survival benefits, but highlighted the persistent absence of therapies capable of regressing existing fibrosis [7]. Recent bibliometric analysis revealed a rapid increase in studies prioritizing lung-function protection over fibrosis reversal, underscoring the urgent need to deepen our understanding of PF pathogenesis and to identify truly disease-modifying therapeutic targets [5].

Epithelial cells, which line the surfaces of organs and tissues, perform essential functions including barrier formation, secretion, absorption, transport, and sensory perception [8]. Under normal physiological conditions, epithelial cells help maintain tissue integrity and contribute to repair processes through complex regulatory mechanisms. However, the dysfunction of epithelial cells plays a key role in the progression of fibrosis. Epithelial cell damage or abnormal repair responses are recognized as early driving factors in the development of PF [9]. In response to physical, chemical, or pathogenic insults, epithelial cells activate regenerative, inflammatory, and repair mechanisms to restore tissue function [10]. However, under pathological conditions, such as chronic inflammation or repeated injury, epithelial cells may undergo abnormal repair responses, leading to epithelial–mesenchymal transition (EMT). Through EMT, epithelial cells differentiate into fibroblast-like cells, directly contributing to the progression of fibrosis [11,12].

Additionally, the interaction between epithelial cells and immune cells during fibrosis progression has garnered increasing attention in recent studies [13]. Damage to epithelial cells, coupled with dysregulation of their repair mechanisms, is considered a pivotal event in the onset of numerous chronic fibrotic diseases. The injury to epithelial cells, their stress responses, and impaired repair processes may be critical steps in the initiation of fibrosis [14,15,16]. These findings suggest epithelial-centered pathways might be promising clinical therapeutic targets for PF. Consequently, elucidating the mechanisms by which functional changes in epithelial cells occur during fibrosis is essential for the development of novel therapeutic strategies for PF. This review will examine the role of epithelial cells in fibrosis, with a focus on the molecular mechanisms underlying epithelial dysfunction in PF, and will explore current and emerging therapeutic strategies targeting epithelial-centered pathways.

## 2. Epithelial Cell Dysfunction Involved in Pulmonary Fibrosis Process

**Figure 1:**
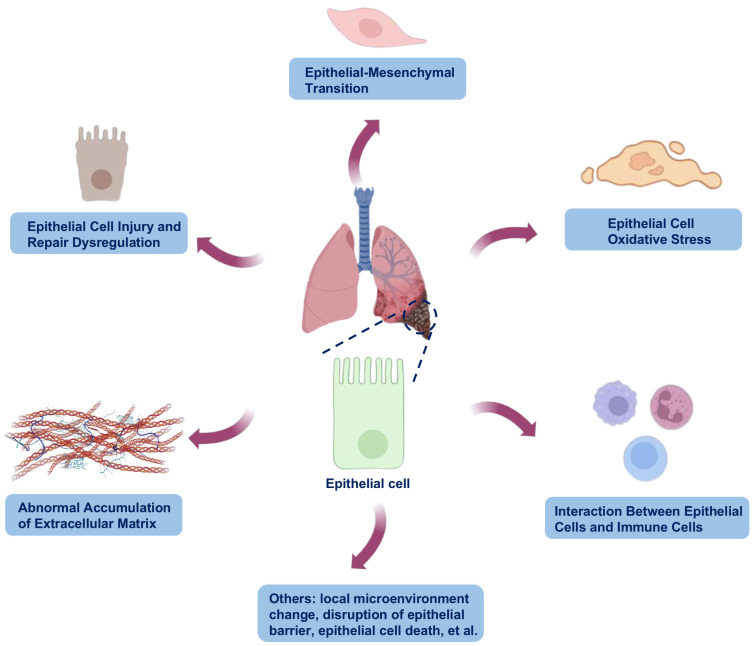
Possible mechanisms of epithelial involvement in the progression of pulmonary fibrosis (PF). In PF, epithelial cells play multifaceted roles in the fibrotic process. They promote fibrosis through various mechanisms, including epithelial injury, epithelial–mesenchymal transition (EMT), pro-fibrotic factors secretion, extracellular matrix (ECM) remodeling, and inflammatory signaling. Damaged epithelial cells lose their ability to repair, leading to destruction of the alveolar structure and triggering fibrotic responses. The images illustrated in the figures were adapted from https://app.biorender.com (accessed on 26 March 2025).

In PF, epithelial cells, particularly alveolar epithelial cells comprising both type I alveolar epithelial cells (AT1) and type II alveolar epithelial cells (AT2), play multifaceted roles in the fibrotic process. These cells not only serve as key initiators of fibrosis but also contribute significantly to its progression. They promote fibrosis through various mechanisms, including epithelial injury, EMT, pro-fibrotic factors secretion, ECM remodeling, and inflammatory signaling (Figure 1). Damaged epithelial cells lose their ability to repair, leading to destruction of the alveolar structure and triggering fibrotic responses [4]. Through these processes, epithelial cells interact with other cell types, such as immune cells, ultimately promoting fibrosis and leading to the aberrant generation of fibrous tissue.

### 2.1. Epithelial Cell Injury and Repair Dysregulation

In the early stages of fibrosis, persistent epithelial cell injury drives the fibrotic process. Damaged epithelial cells lose their barrier function, allowing pathogens and proinflammatory factors to penetrate into deeper lung tissues. These infiltrators then trigger an inflammatory response that activates fibroblasts and immune cells, thereby accelerating fibrotic remodeling [17,18]. Additionally, injured cells secrete pro-fibrotic factors like transforming growth factor-beta (TGF-β), promoting fibroblast activation and ECM deposition, which leads to fibrosis through direct or autocrine/paracrine signaling [18,19,20,21]. In a healthy state, epithelial cells can regenerate and repair tissue, while in fibrosis, persistent injury and chronic inflammation impair this repair process, leading to apoptosis or autophagy [22]. These damaged cells worsen inflammation and fibrosis, leading to irreversible tissue damage [23]. Abnormal autophagy can also cause excessive ECM accumulation, preventing regeneration [24]. Ultimately, the loss of epithelial cell regenerative capacity is a key factor in the irreversible loss of tissue function during fibrosis progression.

### 2.2. Epithelial–Mesenchymal Transition

EMT refers to the transformation of epithelial cells into cells with mesenchymal properties, which is particularly critical in fibrosis and is one of the core mechanisms driving its development. Various cytokines, such as TGF-β, can initiate EMT by inducing epithelial cells to lose their polarity and cell–cell connections, while gaining motility [25,26]. This transition results in the formation of mesenchymal cells, including fibroblasts and myofibroblasts, which possess enhanced migratory and proliferative capabilities and secrete substantial amounts of ECM components, such as collagen. The excessive deposition and stiffening of the ECM not only alter the mechanical properties of the tissue but also further impact cellular activities, thereby promoting the progression of fibrosis [27,28].

### 2.3. Epithelial Cell Oxidative Stress

Oxidative stress promotes fibrosis by affecting cell signaling, inflammation, and ECM remodeling. In the process of chronic injury, the accumulation of reactive oxygen species (ROS) damages epithelial cells, triggers EMT, destroys tissue structure, and forms a vicious cycle of fibrosis [29]. ROS can activate the TGF-β pathway, increase Smad2/3 phosphorylation, and promote epithelial cell activation and ECM accumulation [30]. ROS not only upregulates ECM components expression but also inhibits the activity of matrix metalloproteinases (MMPs). Studies have shown that oxidative stress significantly reduces the activity of MMP-2 and MMP-9, leading to diminished degradation of the ECM, exacerbating tissue stiffness, and promoting fibrosis [31].

NADPH oxidase 4 (NOX4), located on the mitochondrial inner membrane, plays a critical role in PF by generating excessive ROS [32]. ROS accumulation could activate pro-fibrotic pathways such as TGF-β/Smad pathway. This promotes cell proliferation and collagen synthesis, contributing to fibrosis progression [33,34,35]. The pro-fibrotic responses and ECM deposition in the lungs induced by TGF-β1 were inhibited by siRNA-mediated NOX4 knockdown [36]. Deficiency in NOX4 showed protection in mice subjected to bleomycin (BLM) [32]. NOX4 also links to cellular senescence, accelerating the aging of lung cells and exacerbating fibrosis [37,38].

### 2.4. Interactions Between Epithelial Cells and Immune Cells

Epithelial cells interact closely with immune cells, such as macrophages and T cells, which play a crucial role in fibrosis. When damaged, epithelial cells release chemokines like C-C motif ligand 2 (CCL2) and C-X-C motif chemokine ligand 10 (CXCL10), attracting immune cells to the injured area and amplifying the inflammatory response [39]. Studies in mouse models show that this leads to increased infiltration of macrophages and T cells, further enhancing the local inflammatory response [39,40]. Activated immune cells release pro-fibrotic factors like Interleukin-13 (IL-13) and TGF-β, promoting EMT and fibrosis, and creating a positive feedback loop that worsens fibrosis over time [41]. Blocking TGF-β signaling in mouse models has been shown to reduce fibrosis [42]. Additionally, in silica-induced lung fibrosis, epithelial cells in the fibrotic regions can independently synthesize and secrete IgA, which may exacerbate fibrosis by activating fibroblasts [43]. Overall, the interaction between epithelial and immune cells plays a critical role in amplifying the fibrotic response.

### 2.5. Abnormal Accumulation of Extracellular Matrix

During fibrosis, damaged epithelial cells release large amounts of fibronectin, type I collagen, and other ECM components, leading to abnormal ECM accumulation. Normally, ECM degradation and remodeling are balanced by MMPs and their inhibitors, but injury disrupts this balance, causing ECM accumulation and promoting fibrosis [44,45]. Excessive ECM deposition increases tissue stiffness, which activates fibroblasts and worsens fibrosis [46]. Young’s modulus, the ratio of axial stress to axial strain within the material’s linear elastic region, is used to quantify the material’s stiffness under tension or compression. Research shows that fibrotic areas have a significantly higher Young’s modulus, making the tissue stiffer and further activating fibroblasts through mechanosensitive molecules like integrins [47]. The fibrotic ECM not only increases tissue stiffness but also reduces its compliance, thereby restricting alveolar expansion and contraction [48]. This leads to increased respiratory effort, dyspnea, and reduced gas exchange efficiency, causing impaired oxygenation. Additionally, ECM accumulation inhibits tissue regeneration, as seen in cardiac injury, where excessive ECM deposition hampers cardiomyocyte repair [49].

## 3. Key Mechanisms of Epithelial Cells Involved in Fibrosis

### 3.1. TGF-β Signaling Pathway

TGF-β is a key regulator in fibrosis, driving EMT and fibroblast activation, which increases ECM production and promotes fibrogenesis. It is upregulated in fibrosis across various organs, including the kidney, liver, and lungs, through both Smad-dependent and Smad-independent pathways [50,51]. TGF-β1 and its receptors (TGFβRI and TGFβRII) play a critical role in fibrosis progression. TGF-β was also increased in the airway and alveolar epithelium of patients with chronic obstructive pulmonary disease (COPD), revealing the complex and important role of TGF-β signaling in lung fibrosis [52]. In addition to the classical Smad-dependent pathway, TGF-β can also accelerate the fibrotic process through other non-classical pathways, such as the activation of PI3K/Akt and MAPK signaling pathways [53,54]. The amount of evidence suggests that targeting TGF-β cascades has the potential to treat patients with fibrosis.

### 3.2. Wnt/β-Catenin Signaling Pathway

The Wnt/β-catenin signaling pathway plays a pivotal role in regulating epithelial cell injury repair and differentiation. During fibrosis, the Wnt/β-catenin pathway becomes excessively activated. Upon translocation to the nucleus, β-catenin induces the expression of fibrosis-related genes. This upregulation leads to increased protein synthesis and secretion, leading to abnormal ECM accumulation and fibrosis progression [55,56]. In the early stages of fibrosis, the dysregulation of Wnt signaling triggers phenotypic changes in epithelial cells, resulting in EMT and the accumulation of ECM proteins [55,57]. Inhibition of the Wnt/β-catenin pathway significantly reduces the expression of fibrosis-related genes, leading to decreased collagen and fibronectin synthesis, as well as reduced ECM deposition [58]. Moreover, β-catenin inhibition has been shown to suppress EMT and reduce ECM production [59]. These findings highlight the critical role of Wnt/β-catenin signaling in the development of fibrosis.

### 3.3. Notch Signaling Pathway

The Notch signaling pathway plays a key role in regulating cell fate, including activation, proliferation, and differentiation. Its activation has been shown to promote EMT, thus advancing the fibrotic process [60,61]. Specifically, Notch1 and Notch3 activation are closely associated with abnormalities in epithelial cell injury and repair. In PF, Notch1 is a central regulator of AT2 fate, inducing alveolar epithelial proliferation and loss of Napsin A and surfactant proprotein processing, which contributes to fibrosis [62]. Notch inhibitors significantly reduce EMT-related gene expression [61]. In BLM-induced PF, mice lacking Notch1 show reduced fibrosis and collagen deposition, underscoring the role of Notch1 in fibrosis [63]. The activation of Notch3 promotes fibroblast proliferation and differentiation, driving the expansion of the fibroblast population. In contrast, the loss of Notch3 can alleviate PF by affecting the activation of fibroblasts, suggesting that Notch3 is expected to be a therapeutic target for PF [64,65].

### 3.4. Crosstalk Between Epithelial Signaling Pathways in Pulmonary Fibrosis

**Figure 2:**
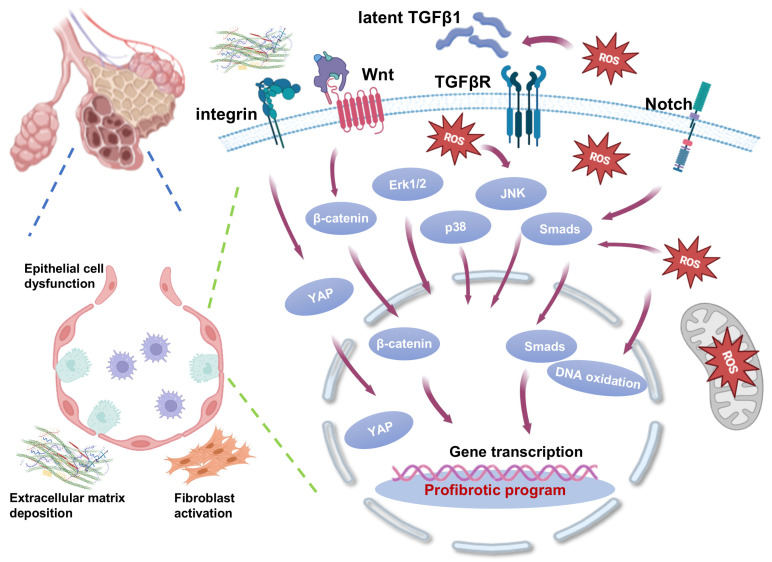
Crosstalk between epithelial signaling pathways in pulmonary fibrosis (PF). In the pathogenesis of PF, multiple signaling pathways work together to drive disease progression through complex crosstalk and synergistic regulatory networks, which interact at multiple levels, including the receptor level, intracellular signal nodes, and epigenetic level, and ultimately lead to continuous amplification of pro-fibrotic signals and irreversibility of pathological remodeling through dynamic network regulation. The images illustrated in the figures were adapted from https://app.biorender.com (accessed on 27 March 2025).

In the pathogenesis of PF, multiple signaling pathways work together to drive disease progression through complex crosstalk and synergistic regulatory networks. These pathways interact at multiple levels, including receptor binding, intracellular signaling nodes, and epigenetic modifications, forming an interconnected network rather than acting alone. Through this dynamic network regulation, pro-fibrotic signals are continuously amplified, driving irreversible pathological remodeling (Figure 2). The cascade amplification effect of this signal network suggests that the therapeutic strategy for a single target may have limitations, and it is necessary to analyze the key network nodes from the perspective of systems biology for combined intervention.

TGF-β and Wnt signaling pathways interact closely, particularly in fibrosis development. TGF-β induces Wnt ligand expression (e.g., Wnt5a, Wnt7b) through Smad3 phosphorylation, increasing their protein levels [66]. TGF-β1 has been shown to synergize with Wnt/β-catenin signaling pathway to induce EMT [67]. Wnt/β-catenin activation inhibits GSK3β, stabilizing Smad3 and forming a Smad3/β-catenin complex [68]. These pathways promote the expression of EMT core transcription factors (Snail and Slug) while inhibiting the expression of E-cadherin [69]. This cooperation between TGF-β and Wnt signaling provides insights for future therapeutic approaches.

Notch signaling exacerbates epithelial damage and EMT, working with TGF-β pathways to drive fibrosis [70]. It promotes the expression of key fibrotic factors, enhancing EMT and ECM accumulation. Notch induces myofibroblast differentiation of alveolar epithelial cells via the TGF-β-Smad3 pathway [71]. Studies show that Notch inhibitors can reduce fibrosis induced by TGF-β, highlighting the role of Notch in TGF-β-mediated fibrotic responses [72]. Additionally, Notch signaling influences fibroblast and myofibroblast activation, leading to ECM remodeling [73]. The crosstalk between Notch and TGF-β pathways represents a critical regulatory axis in fibrosis, suggesting that targeting both pathways may provide a more effective therapeutic strategy.

In fibrosis development, a positive feedback loop between oxidative stress and TGF-β signaling plays a critical role across various organs. TGF-β promotes mitochondrial ROS production by upregulating NOX4 and exacerbates oxidative stress, which in turn activates intracellular pathways driving fibrosis [74]. ROS also plays a bidirectional role in TGF-β signaling. Oxidative stress inhibits Smad7, a negative regulator of TGF-β signaling, leading to persistent phosphorylation of Smad2/3. Under normal conditions, Smad7 inhibits excessive TGF-β signaling. However, ROS accumulation reduces Smad7 levels, diminishing its inhibitory effect, which in turn prolongs Smad2/3 activation and then enhances fibroblast proliferation, migration, and ECM deposition, further promoting fibrosis [51,75,76,77]. This positive feedback loop between ROS and TGF-β signaling reveals the critical role of ROS in the progression of fibrosis. Targeting oxidative stress or disrupting this feedback loop may offer novel therapeutic strategies for fibrosis-related diseases.

Oxidative stress and Notch signaling interact through complex molecular mechanisms, in which ROS plays a crucial role in activating the Notch pathway. Mitochondrial-derived ROS activate the JNK signaling pathway, which subsequently induces the expression of the Notch ligand Jagged1, a ligand that activates Notch receptors [72,78]. Additionally, the intracellular domain of Notch competes with Nrf2 for binding to the coactivator CBP, thereby suppressing the expression of antioxidant genes, such as Superoxide Dismutase 2 (SOD2) [70,79]. Functionally, the ROS–Notch axis drives the emergence of a pro-fibrotic phenotype in epithelial cells, which further promotes fibrosis [80,81]. This interaction between oxidative stress and Notch signaling highlights a critical mechanism in fibrotic diseases and suggests potential targets for therapeutic intervention.

The Hippo-YAP pathway interacts with the TGF-β and Wnt signaling pathways through complex molecular mechanisms, regulating processes such as cell proliferation, differentiation, and fibrosis. Mechanical stress and ECM stiffness inhibit the Hippo pathway via integrin-FAK signaling, leading to the nuclear translocation of YAP/TAZ. Once in the nucleus, YAP/TAZ form a complex with Smad2/3, enhancing the efficiency of TGF-β signaling. Additionally, YAP and β-catenin cooperate to bind to TEAD/TCF elements, activating EMT-related genes such as VIM and ZEB1 [82,83,84]. This cross-talk between Hippo-YAP, TGF-β, and Wnt pathways plays a crucial role in regulating fibrosis and EMT, providing insights for potential therapeutic strategies.

### 3.5. Metabolic and Epigenetic Regulation: A Synergistic Axis in Pulmonary Fibrosis

**Figure 3:**
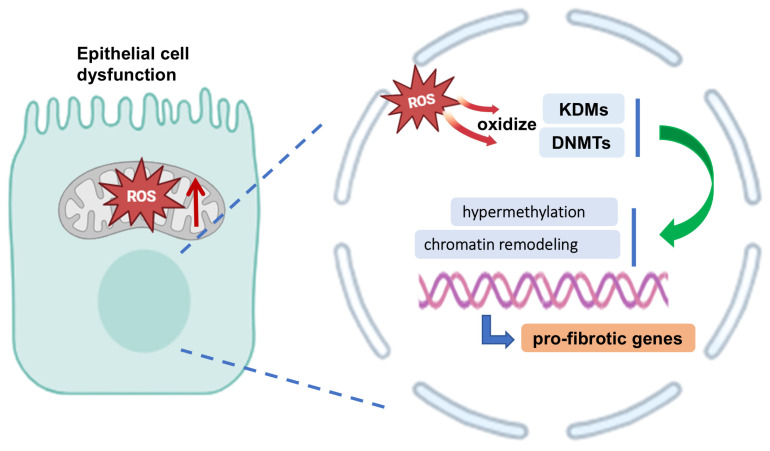
The changes in DNA methyltransferases (DNMTs) and histone demethylases (KDMs) affected by ROS affect the function of epithelial cells and affect pulmonary fibrosis (PF). In PF, dysfunctional epithelial cells undergo profound molecular rewiring to adopt pro-fibrotic phenotypes. Mitochondrial dysfunction generates excessive ROS, which oxidize DNMTs and KDMs, leading to locus-specific hypermethylation and chromatin remodeling at pro-fibrotic genes. The images illustrated in the figures were adapted from https://app.biorender.com (accessed on 27 March 2025).

Emerging evidence highlights the critical interplay between metabolic reprogramming and epigenetic modifications in the pathogenesis of PF. In this process, dysfunctional epithelial cells undergo profound molecular rewiring to adopt pro-fibrotic phenotypes (Figure 3). Mitochondrial dysfunction generates excessive ROS, which oxidize DNA methyltransferases (DNMTs) and histone demethylases (KDMs), leading to locus-specific hypermethylation and chromatin remodeling at pro-fibrotic genes, such as CTGF [85,86,87]. Concurrently, the accumulation of succinate stabilizes HIF-1α, promoting glycolytic flux and increased lactate production, which are key metabolic adaptations in fibrotic cells [88,89].

Additionally, metabolic intermediates like α-ketoglutarate (α-KG) and S-adenosylmethionine (SAM) regulate DNA methylation. AT2 cells with DNMT3A increased exhibit hypermethylation at regeneration-related genes, impairing regenerative potential and promoting fibrotic processes [90,91]. Therapeutic strategies targeting this axis, such as DNMT inhibitors and succinate receptor antagonists, have shown promise in preclinical models by reducing collagen synthesis and normalizing histone lactase, respectively [92,93,94]. Future research should focus on elucidating the spatiotemporal regulation of metabolic-epigenetic crosstalk and evaluating the potential of combination therapies to improve therapeutic outcomes in fibrosis treatment.

## 4. New Technologies Reveal the Heterogeneity and Functional Diversity of Epithelial Cells

The application of cutting-edge technologies such as single-cell sequencing, spatial transcriptomics, and metabolomics has deepened our understanding of epithelial cell behavior in PF and laid a theoretical foundation for better clinical intervention.

### 4.1. Heterogeneity Map of Pulmonary Epithelial Cells

#### 4.1.1. Regenerative Function Partitioning of AT1/AT2 Cells

Alveolar epithelial regeneration under normal physiological conditions is primarily driven by AT2 cells, which act as stem cells and repair the lung by differentiating into AT1 cells. However, during PF, the regenerative function of AT2 cells becomes spatially and state-dependent, leading to impaired repair and altered cellular behavior. In the homeostasis zone, distant from the fibrosis lesions, resting AT2 cells maintain low-level proliferation through Notch signaling, primarily contributing to surfactant synthesis [95]. In the injury response zone, at the periphery of the lesion, AT2 cells are activated into a transitional KRT8+ state, showing both proliferation and TGF-β1 secretion. Under mechanical stress induced by increased ECM stiffness, some cells become locked in a pro-fibrotic phenotype (SFTPC+/SCGB3A2+), losing their ability to differentiate into AT1 cells. In the terminal disruption zone, at the core of the lesion, AT1 cells (AGER+/HOPX+) undergo apoptosis or senescence due to mitochondrial dysfunction caused by abnormal shear forces (ROS increase), leading to alveolar collapse [96,97,98]. In this process, residual AT1 cells exacerbate M2 macrophage polarization by secreting IL-33, thereby promoting the progression of fibrosis [99]. Single-cell multi-omics analysis showed that the Wnt/β-catenin pathway was specifically activated (increased nuclear translocation rate of β-catenin) in transitional AT2 cells, which synergistically drove EMT [100].

#### 4.1.2. Double-Edged Basal Cells in Pulmonary Fibrosis

In normal repair, basal cells differentiate into ciliated cells or goblet cells through the Notch signaling pathway, maintaining airway epithelial homeostasis. Upon injury, basal cells activate the Wnt/β-catenin pathway, rapidly proliferate, and form KRT8+ transitional cells, which differentiate into AT1/AT2-like cells to participate in alveolar repair [101,102]. In the pathological process of IPF, basal cells show high heterogeneity in different phenotypes [103]. In the context of fibrosis, under chronic injury, basal cells respond to ECM stiffness through the YAP/TAZ signaling pathway, converting into a KRT17+ pro-fibrotic subpopulation, inducing fibroblast proliferation and collagen production [104,105]. A recent single-cell RNA sequencing revealed an aberrant basaloid cell type with high expression of pro-fibrotic markers and EMT evidence, localizing to the epithelial layer surrounding the fibroblastic foci [100,106]. WNT7A derived from basal cell or basal-like cells can be captured by neighboring fibroblasts and AT2 cells, promoting fibrogenesis at the fibrotic niche in IPF [107].

In addition, IL-13 drives reprogramming through STAT6 to induce the expression of chemokines, which in turn recruit inflammatory cells such as Th2 cells and eosinophils to form a chronic inflammatory microenvironment [108,109,110]. Epigenetic studies have shown that, in the process of fibrosis, the promoter region of EMT-related genes in pro-fibrotic basal cells is demethylated, which enhances their transcriptional activity and promotes the EMT process, thereby aggravating fibrosis [4,111,112]. Thus, reversing the pro-fibrotic phenotype of basal cells can be a therapeutic target in the progression of PF. DNA methyltransferase inhibitors, such as 5-azacytidine, can reverse the abnormal methylation of pro-fibrotic genes, inhibit the activation of fibroblasts and collagen deposition, and thus slow down the progression of PF [113,114]. However, its role in the regulation of basal cell phenotype still needs to be further verified.

### 4.2. Spatial Microenvironment-Driven Functional Polarization

The spatially heterogeneous microenvironment in PF remodels epithelial cell behavior through mechanical stress, metabolic gradients, and immune–epithelial interactions. Increased ECM stiffness mechanotransduces via YAP/TAZ activation in AT2 cells, promoting their EMT through enhanced nuclear localization of YAP and upregulation of EMT genes [115,116,117].

Succinate, a tricarboxylic acid cycle intermediate, accumulates in IPF and engages its receptor GPR91 to activate ERK and stabilize HIF-1α, driving fibroblast activation [118,119]. Pharmacological inhibition of succinate dehydrogenase attenuates TGF-β1-induced succinate elevation and alleviates fibrosis, whereas exogenous succinate exacerbates it [119,120]. Lactate produced during inflammation induces histone lactylation in macrophages, upregulating pro-fibrotic gene expression and reinforcing fibrogenic signaling [121,122,123,124].

Multiplexed CODEX imaging reveals enrichment of M2-polarized macrophages at fibrotic lesions, where they suppress AT2 cell regeneration via the PD-1/PD-L1 axis and secrete IL-13 to activate fibroblasts and promote collagen deposition [2,125,126]. These findings underscore the complex interplay of mechanical, metabolic, and immune factors driving fibrosis.

### 4.3. MicroRNAs Regulation

MicroRNAs (miRNAs) are crucial regulators of gene expression in fibrosis. Notably, miR-21 is significantly upregulated in mouse models of PF, enhancing TGF-β signaling by inhibiting the antifibrotic factor Smad7 [127]. Treatment with miR-21 inhibitors leads to a significant decrease in collagen deposition and a rebound in Smad7 expression levels, further confirming the pro-fibrotic role of miR-21 [128]. On the other hand, members of the miR-200 family protect against fibrosis by inhibiting EMT-related factors like ZEB1 and ZEB2. Overexpression of miR-200b in mice decreases collagen I levels and increases E-cadherin expression, suggesting that miR-200b mitigates fibrosis by inhibiting EMT [129]. Conversely, miR-200 knockout models show increased EMT factor expression and collagen deposition, highlighting the protective role of miR-200 in fibrosis suppression [130].

### 4.4. Clinical Translation Potential of the Heterogeneity Map

The epithelial cell heterogeneity atlas in PF has significant clinical value in diagnosis, targeted therapy, and prognostic monitoring. Single-cell RNA sequencing and spatial transcriptomics have identified disease-specific epithelial subpopulations, such as SCGB3A2+ pro-fibrotic AT2 cells, leading to novel biomarkers like serum MMP7 and sputum KRT17 mRNA. Combined with AI-based radiomics models, diagnostic accuracy has reached 92%. Targeted therapies, including Galectin-3 inhibitors (GB0139) and YAP/TAZ inhibitors (VP-001), are in clinical trials. Dynamic profiling, such as liquid biopsy, further advances precision medicine. However, challenges remain, including high sequencing costs, complex microenvironment dynamics, drug delivery issues, and lack of biomarker standardization. Innovations in technology and optimized clinical strategies offer promise for accelerating the clinical application of heterogeneity maps and providing more precise treatments for PF patients.

## 5. Anti-Pulmonary Fibrosis Drugs Targeting Epithelial Cells

As mentioned above, alterations in epithelial cell function play an important role in PF. Regulating the injury response of epithelial cells, reducing ECM deposition, and affecting the fibrosis process can effectively promote anti-fibrosis outcomes. In this section, we provide a review of current antifibrotic agents that target the regulation of epithelial cell function in the context of PF. Many new drugs are currently in clinical trials and are expected to provide more treatment options for PF.

### 5.1. FDA-Approved Drugs for Clinical Use

#### 5.1.1. Pirfenidone

Pirfenidone exhibits antifibrotic, anti-inflammatory, and antioxidant properties, making it a broad-spectrum agent for fibrosis. Although its exact mechanism is not fully understood, it influences EMT by inhibiting pro-fibrotic pathways such as TGF-β and fibroblast growth factors, alleviating fibrosis caused by epithelial injury. Pirfenidone inhibits TGF-β1-induced fibroblast activation and collagen synthesis, confirming its role in suppressing EMT [131]. It also reduces inflammatory factors like TNF-α, IL-6, and IL-1β in animal models, and decreases lung tissue inflammation, demonstrating its anti-inflammatory effects [132]. Furthermore, pirfenidone reduces oxidative stress markers (ROS and MDA) and increases antioxidant enzyme activity (SOD and CAT), boosting cellular antioxidant capacity [133,134].

In the CAPACITY and ASCEND clinical trials, pirfenidone significantly slowed the decline in lung function in patients with IPF. Patients receiving pirfenidone treatment exhibited a significantly lower rate of decline in FVC compared to the placebo group, indicating its effectiveness in clinically delaying disease progression [135].

#### 5.1.2. Nintedanib

Nintedanib is a multi-targeted tyrosine kinase inhibitor that blocks pro-fibrotic pathways such as VEGF, PDGF, and FGF, reducing epithelial cell damage, apoptosis, and fibrosis. Its primary action is to inhibit fibroblast proliferation and migration, but it also regulates epithelial cell function to alleviate PF [136,137]. Research shows that nintedanib reduces apoptosis in alveolar epithelial cells and increases E-cadherin expression, supporting epithelial integrity [137]. In preclinical IPF mouse models, it significantly reduced PF, collagen content, and improved lung function and gas exchange [138]. Additionally, nintedanib has anti-inflammatory effects, inhibiting TNF-α and IL-1β secretion, and reducing inflammatory cell infiltration by blocking the PI3K/Akt/mTOR pathway [139]. Approved for IPF treatment, nintedanib effectively slows disease progression and improves lung function.

### 5.2. Drugs Developed Based on Molecular Mechanisms

#### 5.2.1. TGF-β Signaling Pathway Inhibitors

TGF-β is a key factor in PF, promoting fibroblast activation and EMT. Inhibiting the TGF-β signaling pathway can reduce the transition of alveolar epithelial cells into fibroblasts, alleviating fibrosis. Drugs targeting TGF-β, such as antisense oligonucleotides or monoclonal antibodies, block its binding to receptors or inhibit downstream signaling (e.g., Smad phosphorylation), effectively reducing fibrosis caused by epithelial cell damage [140]. For example, tranilast suppresses the TGF-β/Smad2 pathway, and (S)-ibuprofen-pirfenidone conjugate 5b reduces lung histopathological changes and collagen deposition in fibrosis models [141]. While TGF-β inhibitors are still in clinical trials, they have not yet been approved for treating PF.

#### 5.2.2. Antioxidants

Oxidative stress is a significant cause of damage and apoptosis in alveolar epithelial cells. Therefore, inhibiting oxidative stress can effectively block the progression of fibrosis. Antioxidants, such as N-acetylcysteine (NAC), can reduce oxidative damage by scavenging free radicals, thereby protecting alveolar epithelial cells and slowing the progression of fibrosis [142,143]. NAC has shown some efficacy in certain patients with PF, but further research is needed.

#### 5.2.3. Wnt/β-Catenin Signaling Pathway Inhibitors

The Wnt/β-catenin pathway is essential for epithelial repair, but excessive activation is linked to fibrosis. Inhibiting Wnt signaling can reduce EMT and slow fibrosis progression. Currently, Wnt inhibitors are in preclinical or early clinical stages, with no approved drugs. IWP-2 is a small molecule that inhibits β-catenin by interfering with Wnt signaling, showing potential antifibrotic effects in PF models [144]. C59 also inhibits the Wnt pathway by blocking β-catenin translocation and gene expression, suppressing fibrosis in liver and lung models [145]. Additionally, low-molecular-weight Fucoidan from sea cucumber Acaudina Molpadioides exhibits anti-fibrotic activity by modulating Wnt/β-catenin signaling [146]. However, recent studies suggest selective activation of Wnt may also benefit IPF [147]. These findings underscore the potential of Wnt/β-catenin as an antifibrotic target, with future studies needed to confirm their efficacy in specific diseases.

#### 5.2.4. FGF Receptor Inhibitors

The FGF family plays a significant role in PF, particularly in promoting the repair and regeneration of epithelial cells. However, excessive FGF signaling can lead to fibrosis [148]. FGF receptor inhibitors work by inhibiting the overactive signaling pathways, thereby reducing the progression of fibrosis. Fibroblast growth factor-10 (FGF-10) is crucial for the regeneration of alveolar epithelial cells. FGF-10 analogs can promote the repair and regeneration of damaged epithelial cells, thus preventing the progression of fibrosis [149,150]. Currently, FGF-10 analogs have shown promise in animal models, but studies in humans are still ongoing.

### 5.3. New Targeted Drugs Developed by New Technology Applications

#### 5.3.1. Galectin-3 Inhibitors

Galectin-3 is a glycoprotein that plays a crucial role in PF, particularly in mediating interactions between epithelial cells and fibroblasts. Its involvement in PF has attracted significant attention in recent years. Inhibiting Galectin-3 has been shown to disrupt the transmission of fibrotic signals and mitigate the fibrotic response following epithelial cell injury [151]. Currently, several inhibitors targeting Galectin-3 are in clinical trials, offering promising potential for therapeutic intervention.

GB-0139 is an oral Galectin-3 inhibitor developed by Galecto Biotech and is currently undergoing clinical trials to evaluate its efficacy and safety in patients with IPF. Preliminary results indicate that GB-0139 can significantly lower levels of Galectin-3 and may positively impact the progression of PF [152,153]. GR-MD-02, developed by Galectin Therapeutics, is primarily aimed at treating liver fibrosis and other fibrosis-related diseases. This recombinant Galectin-3 inhibitor has shown potential in clinical trials for reducing fibrotic lesions, particularly in patients with chronic liver disease [154,155]. TD139 is another Galectin-3 inhibitor currently under clinical investigation to assess its effects on COPD and PF. Early studies indicate that TD139 has good tolerability and can significantly reduce biomarkers associated with fibrosis [156]. The research on these inhibitors highlights the potential of targeting Galectin-3 as a therapeutic approach for fibrosis-related diseases. However, further clinical trial data are necessary to confirm their long-term efficacy and safety.

#### 5.3.2. MicroRNAs Regulators

Certain miRNAs, such as miR-21 and the miR-200 family, have been identified as key regulators in EMT and fibrosis [157,158,159,160]. These miRNAs play crucial roles in the initiation and progression of fibrosis by modulating various cellular processes, including cell proliferation, migration, and ECM deposition. Interestingly, manipulating miRNA expression, either through inhibitors or enhancers, has emerged as a promising strategy to modify the behavior of epithelial cells and potentially prevent or reverse the development of fibrosis. For instance, miR-29 mimics, such as MRG-201 and MRG-229, have shown significant potential in reducing collagen secretion and maintaining the integrity of lung alveolar architecture, offering a potential therapeutic approach for fibrotic diseases [161,162]. While miRNA-based therapies are still under active investigation, early data suggest that these agents could serve as effective tools for treating a range of fibrosis-related conditions. However, further research and clinical trials are required to fully assess their safety, efficacy, and long-term impact on fibrotic diseases.

#### 5.3.3. Therapies Targeting Epithelial Cell-Derived Exosomes

In response to injury, epithelial cells release exosomes (small extracellular vesicles rich in pro-fibrotic mediators, miRNAs, and proteins) that facilitate intercellular communication and contribute to PF progression. These exosomes promote EMT, activate fibroblasts, and enhance ECM deposition [163]. Notably, miR-21 in exosomes was shown to modulate the TGF-β pathway to further amplify fibrotic signaling [164,165]. The inhibition of exosome secretion with drugs such as GW4869, a neutral sphingomyelinase inhibitor, has been effective in reducing PF in animal models by interfering with pro-fibrotic signaling between epithelial cells and other cells [166,167,168,169]. Although still in the early stages of development, exosome-targeted therapy represents a promising therapeutic avenue for PF and warrants further clinical investigation.

### 5.4. Drugs Designed Based on AI

AI has shown great potential in the design of anti-fibrotic drugs. Through machine learning and deep learning technologies, it can accelerate target discovery, molecular screening and optimization, drug repurposing, and clinical trial design. AI platforms such as Insilico Medicine, BenevolentAI, and Atomwise have made progress in the development of anti-fibrotic drugs. They have identified several potential candidate molecules and optimized their effects through computational simulations and data analysis.

Additionally, AI can be used for drug repurposing, discovering the therapeutic potential of existing drugs for fibrosis. Although this field is still in its early stages, with the continuous development of technology, AI is expected to accelerate the discovery of anti-fibrotic drugs, providing new therapeutic strategies for diseases such as PF. Insilico Medicine has developed a preclinical inhalation formulation candidate, ISM001-055. Inhaled formulations of ISM001-055 achieved high lung exposure and low systemic exposure in preclinical studies and demonstrated anti-fibrotic and anti-inflammatory effects in animal models [170,171]. The ISM001-055 inhalation formulation has good pharmacokinetics and safety, along with good stability and solubility.

### 5.5. Current Challenges

Despite advancements in PF therapies, challenges remain in improving drug delivery efficiency and overcoming resistance mechanisms. Nanoparticle-based drug delivery to lung tissue is influenced by factors such as particle size and the heterogeneity of fibrotic lung tissue, which can result in uneven drug distribution within the lung [172,173,174]. Improved targeting strategies are needed to enhance distribution and therapeutic efficacy. Drug resistance mechanisms, such as the feedback activation of the Notch-Jagged1 signaling pathway, which can drive persistent fibroblast activation, also hinder treatment success [47,72,175]. Targeting these resistance mechanisms through combination therapy or upstream signaling modulation will be critical to improve therapeutic outcomes. In summary, overcoming challenges related to drug delivery and resistance mechanisms is crucial for the development of more effective treatments for PF.

## 6. Future Perspectives

To address the challenges in PF therapy, several innovative strategies are emerging. Advanced technological tools, including cost-effective, high-throughput single-cell platforms and AI-driven data analytics, are facilitating large-scale studies and enabling more accurate predictive modeling. AI and machine learning approaches can integrate multi-omics datasets, identify novel therapeutic targets, and predict patient-specific responses. Notably, AI algorithms, such as those utilizing AlphaFold for the identification of Galectin-3 inhibitors, hold considerable promise in targeting critical mediators of fibrosis progression [176,177,178].

In precision drug delivery, microenvironment-responsive nanocarriers and engineered exosomes provide targeted approaches to modulate epithelial cell behavior. ROS-sensitive nanoparticles, such as those loaded with YAP inhibitors, have demonstrated efficacy in preclinical models [179,180,181]. Additionally, combination therapies targeting multiple pathways, such as TGF-β and YAP/TAZ inhibition, can mitigate resistance mechanisms [182,183]. Epigenetic modulation through DNMT or BET inhibitors presents a novel approach to reversing pro-fibrotic changes [112,184,185]. Personalized medicine will benefit from patient stratification based on single-cell and spatial omics data, guiding tailored treatments like Galectin-3 inhibitors for SCGB3A2+ patients. The integration of digital twin models, combining multi-omics and clinical data, may further optimize individualized therapies.

Although AI-driven drug discovery and spatiomics technologies offer powerful new tools for target identification and biomarker studies, they face significant translational hurdles. AI models rely heavily on comprehensive, unbiased training data, which limits their predictive accuracy for rare diseases and underrepresented patient populations [171,186]. Macromolecular and nanoparticle carriers encounter biologic barriers and macrophage clearance during pulmonary delivery, necessitating extensive in vitro and in vivo validation of their safety in accordance with regulatory guidelines [187,188]. At the same time, spatial omics platforms vary widely in resolution, data consistency, and batch effect control, and the high cost of sequencing and imaging limits their use in large patient cohorts [189,190]. In summary, multicenter validation studies, standardized analytical processes, and prospective biomarker-driven clinical trials are essential to translate these promising approaches into individualized therapy.

Interdisciplinary collaboration across bioengineering, data science, and clinical research is crucial for advancing these innovations. Cross-disciplinary efforts, exemplified by organ-on-chip models, can accelerate drug screening, while global consortia such as the Human Cell Atlas (HCA) and LungMAP are pivotal in advancing biomarker discovery and data sharing.

## 7. Conclusions

This review has comprehensively examined the pivotal role of epithelial cells in the pathogenesis of PF, emphasizing their multifaceted contributions to disease initiation and progression. Compared with the existing literature, our review extends beyond conventional fibrotic signaling pathways by emphasizing the heterogeneity of ECs, including different subpopulations and their specific roles in PF progression. We integrate the functional alterations and potential mechanisms of ECs in the progression of PF with the application of advanced technologies such as single-cell sequencing, spatial transcriptomics, and metabolomics, providing a theoretical basis for better clinical intervention. Additionally, we also explored emerging epithelial-targeted therapies, ranging from FDA-approved agents to novel drugs developed through new technology applications, providing valuable insights into precision anti-fibrosis strategies for clinical translation.

PF is the result of a complex interaction between genetic susceptibility and environmental exposure. Genetic studies have identified rare and common variants, such as mutations in telomerase components and MUC5B promoter polymorphisms, that increase susceptibility to both familial and sporadic disease [191,192,193]. At the same time, environmental factors such as smoking, occupational inhalation of silica or metal dust, pesticides, air pollution, certain drugs, and chest radiation can also lead to repeated epithelial damage and dysregulated repair processes. The role of these factors varies in different patient populations, providing a basis for the heterogeneity in clinical presentation, disease progression, and response to therapy in PF.

Epithelial cells, especially alveolar epithelial cells (AT1 and AT2), serve as both initiators and propagators of fibrosis through mechanisms such as epithelial injury, EMT, oxidative stress, immune cell interactions, and excessive ECM deposition. Dysregulation of these processes, driven by key signaling pathways including TGF-β, Wnt/β-catenin, and Notch, underscores the critical role of epithelial dysfunction in the fibrotic cascade.

Current FDA-approved therapies, such as pirfenidone and nintedanib, have demonstrated efficacy in slowing disease progression by modulating epithelial cell responses and downstream fibrotic pathways. Phase III trials demonstrated that both pirfenidone and nintedanib significantly slowed the annual decline in FVC and reduced the risk of acute exacerbations in patients with IPF [194,195]. In terms of safety, the most common adverse effects of pirfenidone were gastrointestinal symptoms and photosensitivity, while the main adverse effects of ndanib were diarrhea, hypertension and mild elevation of alanine aminotransferase/aspartate aminotransferase [196,197,198]. Small studies have reported reductions in serum Krebs von den Lungen-6 and surfactant protein D with both therapies, but evidence is still limited by small sample sizes and the lack of large prospective cohorts [199,200]. Future studies should focus on biomark-driven personalized dosing strategies to maximize efficacy while minimizing toxicity.

Although both pirfenidone and nintedanib have significant roles in the treatment of PF, their inability to reverse established fibrosis highlights the urgent need for novel therapeutic strategies that target the underlying molecular mechanisms of epithelial dysfunction. Emerging approaches, including inhibitors of TGF-β, Wnt/β-catenin, and Galectin-3, as well as microRNA regulators and exosome-based therapies, show promise in preclinical and early clinical studies. The advent of advanced technologies, such as single-cell sequencing and spatial transcriptomics, has further revealed the heterogeneity and functional diversity of epithelial cells in PF, identifying novel subpopulations and potential therapeutic targets. Additionally, AI-driven drug discovery and precision medicine approaches are poised to accelerate the development of personalized treatments tailored to individual patient profiles.

However, despite the promise of new molecular targets and individualized treatment approaches, preclinical efficacy still relies heavily on rodent models, such as the BLM model. The BLM-induced PF animal model often shows acute inflammation within 1–2 weeks after administration and resolves spontaneously within 3–4 weeks, making it difficult to mimic the chronic and irreversible progression of human IPF. The fibrosis in the model was mostly subpleural patchy, which was significantly different from the typical human usual interstitial pneumonia pattern of honeycomb-like lungs and fibrous foci [201]. In addition, because the experiments were performed in inbred mice between 8 and 12 weeks of age, they do not adequately reflect the advanced age and genetic heterogeneity of IPF patients [202]. To improve translational relevance, humanized precision-cut lung slices can be combined with genetically engineered models to verify key mechanisms and effectiveness.

Despite these advancements, significant challenges remain, including optimizing drug delivery to heterogeneous fibrotic lung tissue, overcoming resistance mechanisms, and translating preclinical findings into effective clinical therapies. Future research should focus on elucidating the spatiotemporal dynamics of epithelial cell behavior in PF, integrating multi-omics data to identify novel biomarkers, and developing combination therapies that target multiple pathways to address resistance and enhance efficacy. Moreover, interdisciplinary collaboration across bioengineering, data science, and clinical research will be essential to bridge the gap between bench and bedside, ultimately improving outcomes for patients with PF.

In conclusion, the heterogeneity of epithelial cells in PF presents both challenges and opportunities for therapeutic intervention. While technological and biological complexities remain significant barriers, emerging innovations in single-cell omics, AI-driven drug design, and precision delivery systems hold great promise. By addressing these challenges through interdisciplinary collaboration and patient-centric approaches, we can unlock the full potential of epithelial cell-targeted therapies, transforming the landscape of PF treatment.

## Data Availability

No new data were created or analyzed in this study. Data sharing is not applicable.
